# Towards a science of global health delivery: A socio-anthropological framework to improve the effectiveness of neglected tropical disease interventions

**DOI:** 10.1371/journal.pntd.0006537

**Published:** 2018-07-19

**Authors:** Kevin Louis Bardosh

**Affiliations:** 1 Department of Anthropology, University of Florida, Gainesville, Florida, United States of America; 2 Department of Environmental and Global Health, University of Florida, Gainesville, Florida, United States of America; 3 Emerging Pathogens Institute, University of Florida, Gainesville, Florida, United States of America; Harvard Medical School, UNITED STATES

## Abstract

**Background:**

Over the last decade, implementation research and a science of global health delivery have emerged as important vehicles to improve the effectiveness of interventions. Efforts to control neglected tropical diseases (NTD) operate in challenging circumstances and with marginalized populations, making attention to context-specific details particularly relevant. Socio-anthropological insights have much to offer a science of NTD delivery. In this paper, an accessible and actionable framework for understanding NTD intervention effectiveness, based on socio-anthropological research, is presented and its utility for program planning and monitoring and evaluation is outlined.

**Methodology/Principal findings:**

The framework was developed inductively by comparatively analyzing three rapid ethnographic studies undertaken in Eastern Africa (2010–2013) on three different large-scale NTD interventions: rabies elimination in Tanzania, sleeping sickness control in Uganda and the prevention of parasitic worms in Zambia. The framework includes five “intervention domains” where the effectiveness of these interventions was negotiated and determined at the local level. This involves: 1) *the terrain of intervention* (including seasonality and geographical variability); 2) *community agency* (including local knowledge, risk perceptions, behaviors, leadership and social pressure); 3) *the strategies and incentives of field staff* (skills, motivations, capabilities and support); 4) *the socio-materiality of technology* (characteristics of intervention tools and the adoption process itself); and 5) *the governance of interventions* (policy narratives, available expertise, bureaucracy, politics and the utilization of knowledge). The paper illustrates the importance of each of these domains by drawing on the case study research, presenting lessons learnt and practical recommendations for how such insights could improve intervention delivery.

**Conclusions/Significance:**

To help close the gap between efficacy and effectiveness in NTD programs, it is important that field staff: 1) generate meaningful knowledge about contextual factors; 2) use this knowledge to tailor field strategies; and 3) create routine mechanisms to account for the dynamic process of implementation itself. The framework presented here offers a simple analytical tool to strengthen these knowledge-to-action relationships existing project planning tools, drawing on the insights of socio-anthropology.

## Author summary

Many efficacious tools exist to control NTDs, but effectively moving these tools and approaches from the boardroom to the village is a complicated socio-political process. In the era of Sustainable Development Goals, global health has become more focused on improving the delivery of existing interventions. Greater attention to implementation research, including the value of social science perspectives, has followed in an effort to build a science of global health delivery. This paper presents an accessible and actionable socio-anthropological framework for understanding the effectiveness factors of NTD interventions. The framework was developed by comparatively analyzing three large-scale NTD interventions in Eastern Africa: rabies elimination in Tanzania, sleeping sickness control in Uganda and the prevention of parasitic worms in Zambia. The framework includes five “intervention domains” where the effectiveness of these interventions was determined: 1) the terrain of intervention; 2) community agency; 3) the strategies and incentives of field staff; 4) the socio-materiality of technology; and 5) the governance of interventions. The paper illustrates the importance of each of these domains, presenting lessons learnt and practical recommendations. As a flexible analytical tool, the framework could be integrated into the planning and implementation process itself, bringing the insights of socio-anthropological approaches into an emerging science of NTD delivery.

## Introduction

It has long been argued that social science perspectives have a great deal to offer the world of global public health. While strides have certainly been made, with the integration of socio-anthropologists and others in research and programs now more mainstream, progress is still slow and uneven [1–3]. This is especially so in countries where neglected tropical diseases (NTDs) are most common. Here, in contexts of poverty and affliction, health system research tends to be down the list of priorities and disciplinary divisions remain more firmly entrenched. At the same time, wealthier scientific and development partners are often focused more on generating evidence for new tools and technologies. The question of how to bring existing interventions to scale, embed them successfully in health systems and ensure they reach their full potential in diverse local settings, across hundreds of millions of people globally, remains somewhat of a twilight zone.

In the era of Sustainable Development Goals (SDGs), implementation research has ascended to the top of the priority list in global health, in light of the emergence of “implementation science” and efforts to create a “science of global health delivery.” While there are a variety of definitions, Allotey *et al*. [4] defined implementation research as:

Applied research that aims to develop the critical evidence base that informs the effective, sustained and embedded adoption of interventions by health systems and communities. It deals with the knowledge gap between efficacy, effectiveness and current practice to produce the greatest gains in disease control.

Against this backdrop, different frameworks are calling attention to how social context and social interaction influence global health implementation. Damschroder *et al*. [5] proposed a meta-theoretical framework of different interacting domains including: how the intervention fits into implementing organizations; the external social and economic environment; the perceptions, abilities and motivations of planners and implementers; and the process of implementation itself: planning, engaging, executing, reflecting and evaluating. Gruen *et al*. [6], drawing on participatory action research, proposed that health programs be viewed as complex social ecosystems where diverse stakeholders interact with very different norms and interests, and where constant engagement, reflection, research and adaptive learning are needed to keep them on track. Frost and Reich [7] proposed what they called the “access framework”, focused on how acceptability, affordability, availability and organizational architecture shape the adoption process for health technologies in resource-poor countries. Importantly, Obrist *et al*. [8] added a focus on livelihood vulnerability and social resilience.

This ethos has also influenced the NTD community; see, for example, *Implementation Research for the Control of Infectious Diseases of Poverty* [9] and *The Global Report for Research on Infectious Diseases of Poverty* [10]. Social scientists have provided important inputs into this agenda, and a new “trans-disciplinary vision” for how to conceptualize the control of diseases of poverty, especially those with more complex disease ecologies, has emerged [11].

According to Prentice [17], ethnographic research in the field of global health has four main principles: it uses fieldwork to build theory, it emphasizes meaning and classification, it explores the negotiated nature of reality, and it emphasizes the central role of context. It can challenge our view of the world and our place in it. Social theories remind us that interventions are dynamic, can have unintended consequences, are socially constructed, involve power dynamics and are sites of negotiation, even contestation, between different social groups [18]. To the ethnographer, interventions are not clean, neutral activities but are complex and messy: a social arena where histories, politics and social conflicts are inevitable.

Anthropological approaches in global health have evolved over the last few decades in response to the push-and-pull of funding streams, scientific networks, methodological innovations and the priorities of program planners and managers, among other factors. These tend to emphasize more rapid methodologies, focused on expedient and actionable forms of knowledge orientated around specific operational questions. This has underpinned the growth of a class of rapid anthropological studies in the 1990s, for example, in WHO-supported childhood diarrheal, malaria, HIV and other programming [12–14]. Studies on NTDs have evolved in parallel to include substantial work on illness categories, drug use patterns, community participation, gender dimensions and community perceptions and responses to interventions [15]. Not all of these, of course, are based on rapid approaches (which have acknowledged pitfalls and risks [13]), and some also draw on action research methodologies, such as participatory rural appraisal (PRA), that use community mapping exercises, diagrams and flowcharts alongside more traditional qualitative methods and participant observation [16], borrowing from the field of international development and humanitarian emergency.

One of the challenges for socio-anthropologists is to bring a deeply contextualized knowledge into the planning and implementation process, in ways that are actionable but not reductionist, taking account of the complexities involved, both from a methodological and social standpoint [19–20]. Monitoring and evaluation (M&E) frameworks and approaches have borrowed from qualitative methods, and many field staff have been trained in these, using them in routine program planning activities. But much of this applied knowledge is based on overly simplistic tools, like the knowledge, attitude and practice (KAP) survey [19]; more flexible and contextualized approaches, like systematic comparative ethnography [20] are a welcomed addition. Despite more biomedical scientists and public health experts recognizing the benefits of more flexible anthropological insights [2], many global health programs still focus predominately on quantitative metrics and struggle with how to conduct, report and operationalize qualitative and ethnographic data.

The aim of this article is to outline a framework for socio-anthropological insights into intervention effectiveness that could, theoretically, assist in orientating M&E and/or an operational research agenda. The framework was developed inductively by analyzing the results of rapid ethnographic studies conducted on three intervention case studies in Eastern Africa (sleeping sickness, rabies and parasitic worms) from 2010–2013. This work focused on understanding intervention effectiveness through issues of coverage, adoption, participation and use of health technologies, and drew on the disciplines of medical anthropology, sociology, science and technology studies, development studies, communication studies and public health.

## Methods

### Description of the three case studies

The three case studies, on which the framework is based, involved three very different health interventions, with the hope that this variability would provide unique insights into how NTD interventions are negotiated at the local level and the various areas where effectiveness is determined. Following an ethnographic approach, the fieldwork relied on mixed methods, combining quantitative data on coverage, uptake and use of health technologies with substantial and in-depth qualitative research, participant observation, document review and ethnographic notes. For a description of the methods see [21], as well as individual peer-reviewed publications in *PLOS NTD* [22], *Medical Anthropology* [23] and *Geoforum* [24]. For greater detail on the case studies, see these individual publications.

There were five major similarities common to these three interventions that provide for strong comparisons and insights. First, they were all “mass interventions” covering large geographical areas and socio-economic contexts that were financially supported by international donors and planned by technical experts. These projects represented “scaled-up” NTD interventions, aimed at targeting hundreds of thousands of farmers and cattle (Uganda), tens of thousands of dogs (Tanzania) and many hundreds of villages (Zambia). Second, the projects had bold targets that aimed for big impact–as noted in their names and goals, they aimed to “eliminate rabies”, “stamp out sleeping sickness” and achieve “total sanitation.” The assumption was that these interventions would showcase the cost-effectiveness and feasibility of preventing NTDs in rural Africa. Third, they relied on local participation, behavior change and the adoption of prevention practices and technologies, such as latrines (Zambia), veterinary insecticides (Uganda) and dog vaccines (Tanzania). These were, in turn, driven by specific justifications that framed this technology as “appropriate” for rural African contexts: rabies vaccination in Tanzania was free and has very minimal side-effects on dogs; community-led total sanitation (CLTS) is an innovative WASH approach, deemed superior to past subsidy-based sanitation approaches in Zambia because it promised to be “community-led” and reliant on locally appropriate building materials; the Stamp Out Sleeping Sickness (SOS) public-private partnership in Uganda aimed to link agro-veterinary business to sleeping sickness control and livestock improvement by creating new systems of veterinary drug delivery that built on existing practices. These were all “low-cost” and “low-tech” health technologies, underpinning the hope that meeting project targets could be achieved within a short period of time with relatively modest funding. Fourth, the delivery of the interventions were done by district and sub-district actors: government staff, local leaders, extension workers, volunteers and private businessmen. Lastly, specific incentive structures were used to mobilize these people and to motivate them to deliver the intervention and to conduct social mobilization, community engagement and risk communication, with the idea that such approaches would be “locally-led” and “sustainable.”

As with any comparison, there were also important differences. The three interventions involved different types of pathogens–sleeping sickness, rabies and parasitic worms–in three different Eastern African countries with diverse cultures, landscapes, languages, politics and other contextual factors. Similarly, the three interventions all used very different approaches (top-down, participatory and market-driven), institutional arrangements (WHO country office, district teams and a public-private partnership), control technologies (vaccination, social mobilization for pit latrines and veterinary insecticides) as well as delivery networks and local incentive structures to enroll support and participation. These are all summarized in Table 1.

**Table 1:** Important differences between the case studies.

Important differences	WHO rabies elimination project, Tanzania [22]	Stamp out Sleeping Sickness, Uganda [23]	Community-led total sanitation project, Zambia [24]
Disease focus	Rabies	Zoonotic sleeping sickness, bovine trypanosomiasis and tick-borne diseases	Sanitation-related diseases, including helminths and cysticercosis
Main target population	Dog-owners	Cattle owners	Open defecators
Locations	Kilombero and Ulanga districts, Southern Region	Dokolo, Kaberamaido, Serere and Soroti districts, Eastern and Northern Region	Katete district, Eastern Province
Approach	Top-down	Public-Private Partnership (PPP)	Community-based and Participatory
Technology	Vaccination	Veterinary insecticides and promotion of the restricted application protocol (RAP)	Social mobilisation and locally-available pit latrine innovations
Incentives for communities	Rabies prevention in dogs and people	Improved animal production, veterinary services and prevention of sleeping sickness	Improved sanitation and community empowerment
Delivery strategy	Government veterinary extension workers	Private veterinarians and animal health workers	Community volunteers
Incentives for implementers	Per diems	Business inputs, trainings and long-term support	Community service and small financial/material benefits
Governance	WHO country office and district veterinary officers (DVOs), funded by BMGF	A consortium of public and private partners in Uganda and Europe, funded by DFID, EU and private philanthropy	District water and sanitation coordinator under local government and supervised by UNICEF, with funding from DFID

Unexpectedly, the individual coverage data found in my large-scale surveys (and validated by my qualitative and observational data) showed that all three of the interventions achieved disappointingly low uptake: 25% vaccination coverage in Tanzania (from a survey of n = 6,157 households), 31% latrine coverage in Zambia (n = 922 households), and an 8.7% market share of the SOS-supported insecticide (known as Vectocid) in Uganda (survey of n = 87 veterinary shops) [22–24]. These projects not only shared these low coverage rates; many of the most salient reasons for why dogs were not vaccinated, latrines were not constructed, and veterinary insecticides were not purchased and used by livestock keepers had many underlining commonalities.

This low coverage was also not inevitable. There were a number of adaptive pathways–as the case studies all make clear below and in the individual case studies [see 22–24]–that could have been used to increase coverage to more acceptable levels both initially and as the interventions progressed over time. Many of these required only modest changes in operational plans, suggesting that the research approach taken here could have substantially improved intervention delivery and population health.

### A Socio-anthropological intervention effectiveness framework

**Fig 1:**
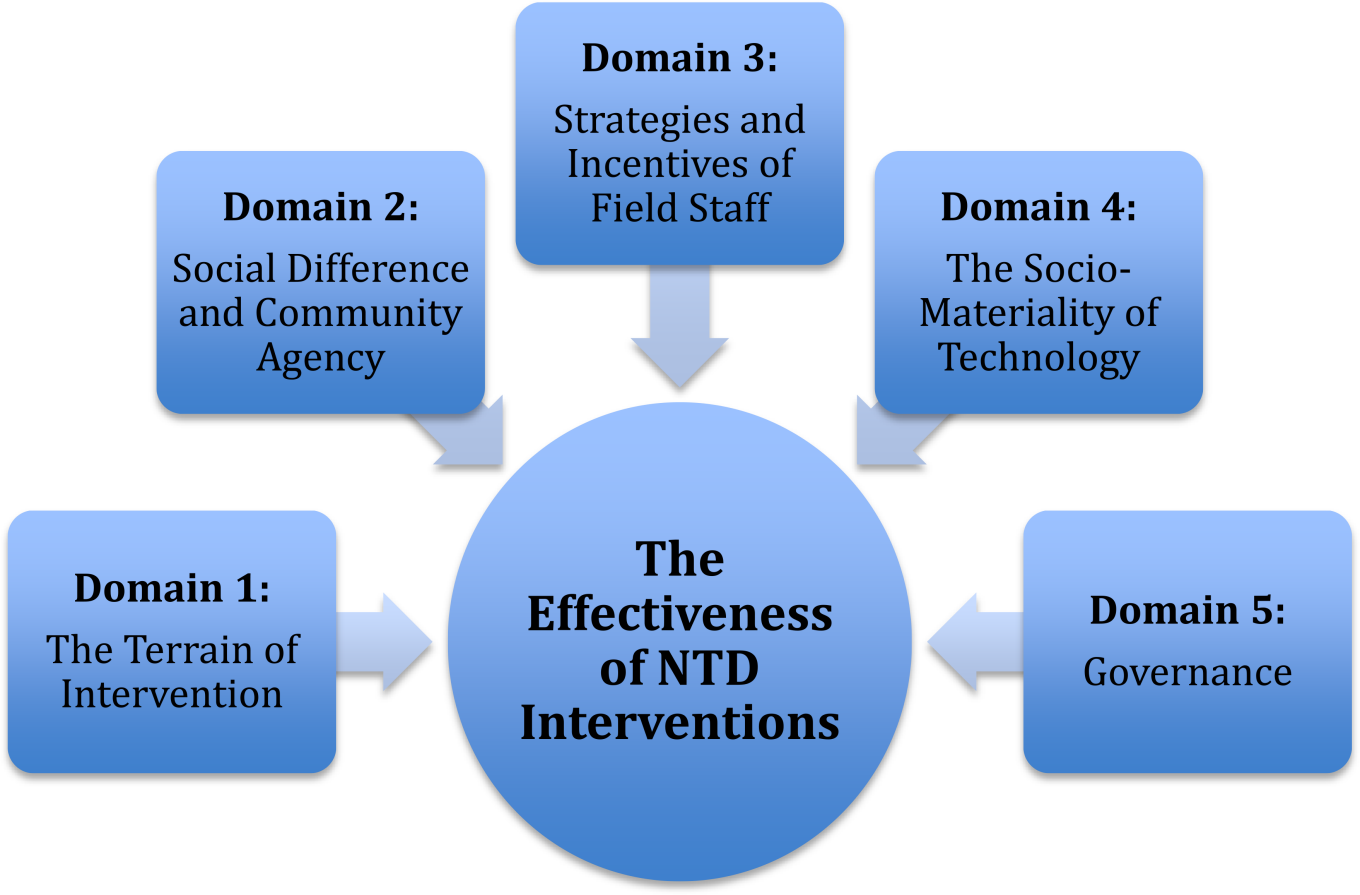
An anthropological framework for NTD intervention effectiveness.

In this paper, a framework for socio-anthropological engagement in NTD intervention effectiveness research and program planning is outlined that draws on a synthesis and analysis of these three case studies. This framework seeks to convey the key areas where effectiveness is negotiated as a complex set of interactions between ecosystems, local communities, animals, implementers, health systems and policymakers, within their broader biosocial context. As shown in Fig 1, this has five “effectiveness domains.” Of course, the framework does not intend to be fully comprehensive; rather, it is presented here as a flexible conceptual tool to assist those planning and implementing NTD control to think about these critical issues.

While these three interventions provided the initial analytical lens for the framework, it is worth noting that my thinking has also been informed by subsequent research and control programs, published on mosquito-borne diseases (lymphatic filariasis, malaria and Zika) in Haiti [25], cysticercosis and helminths in Lao PDR [26], rabies in Indonesia [27], cystic echinococcosis in Morocco [28] and other NTDs [29–30]. Overall, this breadth of experience (of different diseases and contexts) has only strengthened my conviction of the utility of such a framework in assisting to inform NTD control interventions.

## Results

### Domain 1. The terrain of intervention: Seasonality and geographical variation

Global health interventions function over a “local space” where technologies and tools are deployed and issues of coverage and impact are measured and determined. These *terrains* of intervention are created through strategic decisions based on available resources, political expectations and epidemiological knowledge, and aligned with regional, ecological or district boundaries. There is a specified, and unfortunately often all-too-short, time period where the “field” needs to be understood and transformed [31]. Outside the usual realm of “socio-cultural practices”, diverse social groups, governance histories and micro-ecologies exist within these geographies; local livelihoods interact with land-use patterns, seasonal fluctuations, human movement and migration and new socio-economic pressures, many times outside expectation [32]. My research showed that this variability is important but can easily be subsumed by the challenges of creating technical delivery systems.

First, seasonal and geographical variations in local livelihood systems can be in direct conflict with intervention delivery schedules. Low levels of attendance at CLTS sanitation empowerment meetings in Zambia were due to the program starting implementation when farmers were busy harvesting their crops. Seasonal changes in crop farming and livestock management drove the migration of pastoralists in the Tanzanian rabies case study, the social group with the largest number of dogs. However, the elimination program did not account for this–it was one of the most important reasons for the low vaccination rate. The full-scale (and military/police-led) eviction of thousands of livestock-keepers from the Kilombero Valley after my fieldwork, due to concerns about soil erosion and land carrying capacity, demonstrated just how important population movements can be. Planning effective dog vaccination in subsequent years would require accounting for these communities in the wider WHO elimination area. See [33] for a review of this issue in relation to NTDs more generally.

These dynamics clearly have a major impact on intervention coverage, showing that there is often an optimal “window” for interventions in a given geography, orientated around these seasonal changes and the livelihood patterns of high-risk groups. Geographies need to be problematized as diverse and coupled to livelihood-seasonal change instead of the predominate tendency to conceptualize them as uniform and singular.

Seasonality also influences the purchasing power of households. In the Ugandan sleeping sickness case study, reductions in income during the dry season meant that there was little money available to buy insecticides. This happened to correspond with seasonal tick and tsetse population reductions, mitigating an otherwise dramatic influence of seasonality. But it also meant that the veterinary drug shops established by the SOS program struggled, making it harder for these professionals to make a living. The fragility of local economies and livelihoods in the Ugandan study area were also dramatically affected by a foot-and-mouth disease (FMD) quarantine, an epidemic of cassava mosaic disease, severe flooding followed by drought and a longer-term crisis of land fragmentation, due to high population growth. These vulnerabilities led to a volatile market for the veterinary drug sellers and their community-based animal health workers.

Another, perhaps obvious finding, is that intervention staff have a harder time moving along local terrains during the wet season. The large distances, with poor road conditions, created high translation costs for the SOS veterinarians in Uganda, reducing the time they were willing to educate farmers, at least when direct financial support for community-based education was withdrawn by the financing partners. Large distances also dissuaded the animal health workers from spraying cattle–they did not like “running around chasing people” (as they would say)–one reason, among others, for why they preferred the more profitable work in injectable (treatment) drugs.

Seasonal variation and human movement patterns had an impact on local disease risk perceptions and influenced people’s willingness to adopt health technologies. For example, in Zambia, the fact that people defecated more in their agricultural fields than in their latrines (or around their village) in the rainy season, helped to normalize open defecation and de-motivate households to maintain latrines in the village since construction could only address “half our sanitation problem.” Seasonal weather and changing ecology contributed to many latrines collapsing. Villages that were densely settled, due to historic land-use drivers going back to British colonialism, had created bylaws preventing some households from building latrines due to concerns about contamination and miasmic notions of disease spread.

Generally, the distribution of disease is seldom uniform within a certain intervention area, but often clustered in “hotspots.” Interventions have to make strategic spatial decisions regarding delivery and coverage: the placements of central vaccination point in Tanzania, the location of veterinary drugs shops in Uganda and which villages will be triggered for CLTS in Zambia. In the three case studies, delivery was always conceptualized, by planners, at an abstract district-level without adequately considering the social ecology or epidemiology of the area. In Zambia, certain conditions–many of these tied to ecological characteristics (i.e. access to latrine material) and levels of socio-economic development–offered CLTS the best chance of having an impact on sanitation. In Tanzania, the majority of the dog population was found in very remote pastoralist villages, despite the fact that these were not very well covered by the vaccination program. In Uganda, the SOS supported veterinarians did not focus their efforts on villages with active sleeping sickness patients, despite hospital staff (and hospital records) being available in the local treatment hospital (the focus was on meeting monthly sales targets and no shop was actually located in the most highly-endemic sub-county). Just as epidemiologists now speak about “super-spreaders” [34], intervention planners should operate according to targeted strategies that focus on high-priority areas.

These examples all show the importance of diversifying our idea of the “intervention terrain” as we seek to use knowledge to improve implementation, paying attention to local livelihoods, human movement, seasonal change, geographic variability and epidemiological hotspots.

### Domain 2. Social difference and community agency

As with the terrain of intervention, there is also a need to engage with social difference and consider variations in human perspectives, attitudes, logics, social organization, interests and agency. There is a tendency to reify communities as the ultimate target for interventions [35]; but a community is a social construct, a network of relationships and dependencies, and not a geographically bounded unit. Rather, intervention terrains have diverse social groups with differences in wealth, ethnicity, livelihoods, power, knowledge, cultural norms and needs, capacities and constraints. Some people will be more interested than others in the intervention, and more able to adopt technologies and health behaviors. Some will refuse the intervention and may influence others to do the same. Some will recommend changes. This means that planners and implementers need to understand the “public” in public health: they are able to exert influence, make their voices heard and transform plans and policies [36]. But they also have constraints: “throughout the world, those least likely to comply are those least able to comply” [37].

My research found that the intervention adoption process, for insecticides, vaccines and pit latrines, reflected the “uneven playing field” [38] of the rural East African village and deep historical trends of socio-economic and political marginalization. Interventions to alleviate diseases of poverty need to navigate these contexts of destitution, deep inequality and even squalor. The reality is that adoption of health technologies and prevention practices tend to align with higher levels of material wealth and social capital at local level. In Zambia, for example, latrine ownership acted as a symbol of modernity, and it was those households with better education, mobility, access to government subsidies, housing, food security and social networks who build and used them (before and after CLTS). Decades of war, rapid inflation, population growth and other developmental challenges were repeatedly stressed in my Ugandan research to explain why people (sometimes called the “disorganized people”) preferred the cheaper non-tsetse effective insecticides (those only effective on ticks and not the tsetse flies that spread deadly sleeping sickness), despite many knowing that they were inferior products. In Tanzania, canine vaccination was not necessarily influenced by wealth but rather by the motivation for people to keep dogs–dogs that were better cared for and had a defined role in the household were more likely to be vaccinated. Owners were also more able to bring these dogs to the central vaccination point because they were better-behaved dogs. This shows that certain social groups and geographical locations are more likely to be receptive to the intervention.

Motivation to comply with the three interventions, and adopt prevention practices, had a great deal to do with how local people understood the benefits involved. Exposure to “scientific” frames of reference facilitated by veterinary and health extension workers, ideas of social and communal responsibility and a desire to be “modern” were found to play important psychological and cultural roles in all three case studies. Generally, abstract biomedically-defined explanations only went so far in persuading people; rather, what was directly observed, experienced and narrated in locally-understood ways by the community had much more persuasive power. For example in Uganda, many farmers (who were used to spraying insecticides directly onto ticks to kill them) found it hard to understand that cattle sprayed with insecticide could kill a tsetse fly who came to feed on it many hours later. A “hybridism” between experience, local knowledge of disease and biomedical information predominated, something that risk communication and education, which was only done sporadically in these interventions, could have better incorporated.

Local ideas about the value and usefulness of practicing disease prevention strategies were important to people’s motivation, and based on personal experience. In Tanzania, the fear of rabies was frequently mentioned as a motivational force, since people had heard stories (often through their social networks) of previous victims and also feared mass dog culling of non-vaccinated dogs (which local government sometimes implemented on its own accord). The level of input (in time and resources) that people had to invest to rabies control (annual vaccination) was minimal compared to the other case studies, reflecting the lower end of the “participation scale”–as discussed by Rifkin [39]. This certainly helped amplify people’s willingness to comply. In Uganda, experiences of tsetse and ticks and a villages’ location relative to swamps and bushes (breeding areas) were important causes for the level of priority given to sleeping sickness and cattle diseases. Having an animal die from a tick-borne disease was the most often mentioned reason for why farmers began to pay and use insecticides on a regular basis.

De-motivational forces were found to be significant in Zambia and Uganda, where adopting long-term prevention practices were framed as a “gamble” and “risk”, partially because it was never guaranteed to work. This is part of the challenge with adopting health promotion behaviors: they are evaluated based on costs, effort, potential benefits, and in relation to the other multiple vulnerabilities people face. Many simply concluded that the control of neglected diseases was not a priority for them. These were “rare” diseases, and it was better for people to invest their time into something else. This was especially the case when transmission pathways involved more than one route, such as for sanitation-related diseases where one could still get sick despite having a latrine, for example.

An important force behind participation and the promotion of community compliance was the exercise of power, influence and authority at the local level. Punitive efforts included “bylaws” to lock latrine doors and impose “fines” for not having a latrine in Zambia. In Tanzania, this included “village laws” to cull all non-vaccination dogs, dog registration and financial reciprocation laws if a rabid dog attacked someone. Social pressures and simplified local narratives about why people should comply with the interventions were important in normalizing participation and making the case, in locally understandable terms, for compliance and involvement. These included: ticks can kill your cattle; open defecation makes people in the village sick; and not vaccinating your dog can kill your neighbor. These did not necessarily focus on specific diseases, and tended to use stories about local people’s experiences. There was also a different angle to public agency; a household would build a latrine to show their wealth and prestige, to further develop their building techniques and assist in mobilizing (or rebuking) other community members. In this case, those who did not act on these activities were frequently looked down upon, and sometimes mocked and ridiculed as “unsanitary citizens”, even if the ultimate reason was their extreme poverty. Briggs and Mantini-Briggs [40] describe this process in their superb ethnography of the 1990s cholera epidemic among indigenous communities in Venezuela.

Alternatively, all interventions depended on the support and legitimizing labor of local leaders, who were essential in mobilizing community members to attend meetings, spread information and manage aspects of the intervention. In Tanzania, village leadership arranged the location of the central points and disseminated information about the campaign, often door-to-door. In Zambia, poor leadership–where many village leaders themselves, for example, did not have a latrine–contributed to a lack of cohesion and motivation that was only overcome in a few villages with active youth groups, income generation groups and a stronger women leadership culture. A similar case was found in Uganda, where the business aspect of the intervention meant that these leaders only mobilized the community if they were paid a small “motivational” fee.

### Domain 3. The strategies and incentives of field staff

Agency, of course, is exerted not only by the recipients of interventions but also by those charged with implementation [25, 31, 38, 40]. The context of the health system, including aspects like human resources, information systems, drug supply chains, basic infrastructure and the culture of management and care, are incredibly important. Interventions require enrolling the support of actors embedded within these systems, and it is often these health staff, community outreach workers and volunteers that are responsible for actual implementation, translating between the different logics and interests of the intervention and the community [29]. In this process, the interests and relationships of these field staff play an overwhelming role in success or failure.

In the case studies, the delivery and planning of interventions was the task of district bureaucrats, extension workers, local leaders, private shops and volunteers. Outside of the training room and models put forth by outside experts, it was these stakeholders (and their working norms, cultural knowledge, incentives and motivations) that shaped the course of events through field-level decisions and social encounters. In Zambia, for example, many of the local champions trained by the CLTS project used the approach in a piecemeal fashion: they did not use the word “shit” (which is used to provocatively illustrate the fecal-oral-disease route) and rarely conducted any follow-up visits with village groups. But they were also clearly not very competent: many were not very well respected by these communities, lacked technical knowledge about latrine construction itself and could not record data properly. In response, people were reluctant to attend community meetings and voiced expectations for hardware subsidies (instead of following the community-driven ideology of CLTS). While local environmental health technicians (EHTs), employed by the Zambian Ministry of Health, were to oversee these volunteers, they were never given any clear expectations, resources or guidance in how to do so. These EHTs themselves operated under severe staff shortages (I often observed clinic cleaners acting as doctors and nurses at these local health centres), corruption, inadequate supplies and low morale.

In Tanzania, veterinary workers were tasked with selecting the central point and mobilizing dog-keepers (in collaboration with village leaders), ensuring adequate supplies of vaccines and administering the vaccines. Although they often enrolled the support of local leaders, I found that many of the vaccine central points were placed in locations close to major roads, far away from the more remote (and difficult to access) areas where, unfortunately, there were more dogs. Information dissemination to these rural locations was limited.

As Pigg [41] has argued in relation to traditional birth attendants in Nepal, the tendency for projects to categorize local actors involved in implementation based on overarching stereotypes can create simplistic assumptions, leading to inappropriate delivery structures. Staff may need to be fired and replaced, making staff quality control and regular team meetings an important component of an effective intervention system.

Many of the volunteers in Zambia had been selected by local political allies with the expectation of gaining monetarily from the program, without doing any serious work. They were provided bicycles and promised money for achieving a set number of Open Defecation Free (ODF) villages but had little oversight, provision of material (pencils, paper and airtime) and clearly defined benefits rewarding hard work. Motivation and support offered to volunteers has also been noted in the ongoing delivery of ivermectin for the control of onchocerciasis in West Africa, where high dropout rates have revolved around a lack of incentives and supervision, long travel distances, other livelihood duties, drug supply problems and working in areas not familiar to the volunteers–volunteers have also been noted to perform better where money has been provided, such as in polio vaccination [42–43].

Field staff have expectations that need to be met to ensure their continued enrolment and performance. For them, global health interventions are a source of work, of salary, prestige and livelihood–as described by Geissler [31] in his ethnographic work in Kenya. The insecticide sprayers supported by the SOS vet shops in Uganda, for example, expected regular workshops, training, subsidies, drugs on credit and various types of free materials such as spray pumps, overalls and gumboots. But these were not provided, at least as often as they assumed would be the case, which reduced their commitment to project goals. Local leaders in Tanzania expected some small financial incentives for mobilizing farmers and livestock keepers in remote areas. In some cases, these were not provided, and coverage in these areas was considered lower than others.

### Domain 4. The socio-materiality of technology

When we speak about interventions, we are also talking about technology. The ways that technology characteristics and features embed, and are embedded within, social relationships offers an interesting conceptual approach to explore how global health interventions are delivered. We can call this approach *socio-materiality* or the “social life” of technology [44–46].

The three case studies showed how the technologies themselves mediated adoption, delivery and use patterns. In Tanzania, rabies vaccines required cold-chain storage that needed to be delivered at specific central points and whose supply depended on international procurement and adequate syringes. Preferences for non-tsetse insecticides in Uganda involved the characteristics of the insecticides and the effect of the drug: the smell, color, packaging, residual period and mode of action. The fact that many farmers only used insecticides to target tick predilection sites was responsible for the continued preference for amitraz products, which are not effective on tsetse flies, only ticks. To use the insecticides, farmers had to buy or borrow spray equipment (which often broke) and needed protective gear. Without them, they used water bottles, which meant that dilution rates and application methods were not ideal.

A different scenario presented itself in Zambia. Weather and insects destroyed poorly constructed latrines while the level of faecal matter in the pit created impressions that they were “unhygienic.” People had to negotiate the landscape as they searched for the more durable logs to construct the latrine base and acquire the necessary material (bricks or bamboo) to make the superstructure; these were increasingly difficult to find in some areas due to land-use pressures and environmental change. Latrine construction was variable, with many different designs. Building was influenced by the technical knowledge of the owner and builder and their relationships. Latrines required maintenance, had different longevities and were spaced away from homes. Access to durable materials and building techniques played a major role in influencing latrine construction, use and maintenance.

Technologies, social forms and ecological characteristics are embedded within a dynamic web of causality. Viewing health technologies as having a socio-material existence promotes understanding these dynamics as an essential step in intervention effectiveness.

### Domain 5. The governance of interventions

A true science of global health delivery cannot simply focus on the deployment of predetermined interventions but should also critically evaluate the appropriateness of these interventions, their policy models and wider political economy. Negotiating bureaucratic procedures, knowledge flows, authority structures and power struggles between difference stakeholders in a postcolonial world are inevitable components, with major repercussions for the planning and implementation process. Interventions are also designed through policy narratives that define problems and solutions in specific ways; but these storylines can be overly simplistic to reduce uncertainties, appeal to ideals of feasibility and to enroll support (see the work of Roe [47] for an overview of this position).

For example in Zambia, the larger policy environment of the Millennial Development Goals (MDGs), local government decentralization reforms, the failure of past latrine subsidy approaches and the need to “reinvent” the sanitation sector as well as a global discourse about the appeal of the CLTS technique itself (its simplicity, low cost and impact) motivated the rationale for its implementation. In Uganda, the use of private veterinarians to sustain sleeping sickness parasite reductions, after mass cattle treatments, was generated through an emergency narrative of the eminent merger of the two sleeping sickness forms (Rhodesian and Gambian Human African Trypanosomiasis (HAT), which is unique to Uganda), the synergies between business and public health and the low cost and simplicity of insecticide application. These cogent narratives were framed as locally appropriate but ended up locking themselves into certain delivery pathways involving a specific set of actors, that was difficult to modify–a pattern discussed at length by Leach et al. [48] in relation to ongoing problems with international development planning and implementation more generally. Hence the narrative helped enroll certain actors and perspectives while excluding or marginalizing others.

Policy processes can drive the mobilization of resources and the arranging of intervention strategies in very linear and technocratic ways, where confidence in the intervention’s social engineering (as the political scientist James Scott in his well-known work, *Seeing Like a State* [49], would say) becomes over-extended. In my case studies, the interventions were funded, managed and driven by different stakeholders–international agencies, academic institutions, the private sector, philanthropic foundations and a variety of government ministries (this institutional ecosystem for NTD control is described in the review paper by [50]). Multiple bottlenecks in the planning, managing and governance of the interventions limited field-orientated pragmatism, most often influenced by various differences between the stakeholders involved and their ability to learn from operational mistakes and maneuver within their spheres of influence.

In Zambia, the focus of UNICEF on strengthening local government decentralization led to CLTS funds and management being channeled through the Ministry of Local Government and Housing (MLGH). However the MLGH rural water and sanitation department had little experience with participatory methods or in rural sanitation. In my study district, the department was staffed by recent graduates from other urban areas of Zambia who had somewhat patronizing views of “dirty villagers”, paid no attention to the past history of sanitation interventions in the district and did not have a strong desire to involve chiefs, EHTs and local volunteers, as emphasized in “true” CLTS field guides. Mismanagement, then, was a key to the low effectiveness of the project, but so were the important institutional histories, norms and conflicts between the different stakeholders. Had the district focal person for CLTS been more committed to success, and been more supported in this regard, outcomes would likely have been very different, as occurred in neighboring districts.

These issues were also encountered in Tanzania and Uganda. The rabies elimination project, centrally organized by the WHO office in Dar es Salaam, distributed equally set budgets to all 28 districts irrespective of geography, infrastructure and dog populations. Kilombero and Ulanga districts, however, were some of the largest districts and a more flexible budget planning approach would have helped address many problems. The fact that the program budget was often sent at unpredictable times of the year, and needed to be used before the end of the fiscal year, obliged the district teams to use the funds when extensive flooding and pastoralist migrations had occurred, as mentioned above. Furthermore, the history of Structural Adjustment Policies (SAPs) (macro-economic reforms instituted by the International Monetary Fund and World Bank in the 1980s as part of a neoliberal agenda) on the veterinary sector in Tanzania meant that staff capacity was sub-optimal, and contributed to negative community perceptions and relationships with local vet extension officers. The top-down methods of planning used by the WHO country office maintained these rigidities.

In Uganda, the interests of the private sector partners meant that the project could not easily adapt to promote a cheaper insecticide. The eventual rolling back of financial support for community education (airtime, money for village leaders, motorcycle repairs and salaries) was driven by the aim of creating self-sustaining businesses. Here was a narrative that sustainable veterinary business could drive sleeping sickness control. But this did not take into account the particularities of disease epidemiology, or the fact that the original spray-team model needed to be adapted. Important changes in socio-demographics and delivery during the course of the SOS interventions were also unaccounted for: a total of 80% of veterinary shops in my four study districts (survey in 2012) had been established since the original business model intervention in 2008. However the changing veterinary drug market, the continued movements of infected cattle into the area and the high amount of non-tsetse insecticides being sold were left unaddressed.

These examples show that policy pathways are hard to change once they are set into motion. A lack of finances, capacity and reflexive management–itself influenced by the conceptual frameworks for action and organizational limitations–all serve to maintain existing courses of action, despite the need for adaptation and fine-tuning on the ground.

There were alternative governance arrangements that could have avoided some of the institutional barriers involved—for example, rabies vaccination managed by NGOs in the Serengeti (Tanzania) were known as more adaptive to local circumstances; the medical sector and International NGOs were both considered more competent to implement CLTS; and not having been tied exclusively to the SOS brand insecticide through private sector partners, or working more closely with the local HAT treatment centers, could have opened up the possibility of promoting cheaper pyrethroid products, and/or tailoring approaches to sleeping sickness endemic villages.

Management inertia was maintained by a lack of accountability, poor monitoring and evaluation systems and an obscuring of the true impact of the program on the ground. This is something that critical medical anthropologists, working in diverse locations around the world, have begun to unpack as they shine their ethnographic gaze on to the bureaucracy and performance of global health itself [51–52]. Intervention ownership was an important factor as well; in all cases, the interventions appeared (and were often spoken about as being) “imposed” by external stakeholders and were time-limited. The short project cycle appears to generate its own psychological burden that negatively influences the drive for success for local managers and field staff: why rock the boat if the project is going to end soon anyways, and it will not necessarily further your career interests? Building up delivery networks and service provision is itself a process that, in many ways, defies the otherwise short-term goals and targets. There are also long-term challenges with capacity, state-citizen relationships, employment and governance that need to also be acknowledged.

## Discussion

The way we think about the world influences the way we approach social problems, like disease and poverty. The word “intervention” itself comes from the Latin, meaning a “coming-between.” In global health and NTD control, intervening is a social and political act, one that extends biomedicine, public health and development (physically and socio-culturally) from the center (where wealth, power and material advancement are greater) to the periphery, where “diseases of poverty”, by their very definition, are predominately found and clustered [53]. The different world of the international boardroom, the district office and the village (or pastoralist field and urban shantytown) offer very different subjectivities. Inefficiencies, poor decisions, blind-spots, conflicts and unspoken social rules mediate the relationships between these social groups. This is a complicated process, especially as the world continues to evolve from colonial pasts into uncertain multi-polar geopolitical futures where interlocking socio-ecological global crises will present unpredictable challenges for NTD control [29–30]. There is a lot to unpack here, as the discussion above has shown, and as a synopsis of the key findings from the three case studies and their relevance to planning and M&E, show in Table 2.

**Table 2:** Key findings and operational insights from the study relevant to NTD intervention planning and implementation.

Effectiveness Domain	Key findings from the three case studies	Some operational lessons
The Terrain of Intervention	- Seasonal and geographical variations in local livelihood systems can be in direct conflict with intervention delivery schedules. - Purchasing power of households and the time and resources they are willing to invest in disease prevention have seasonal patterns. - Seasonal and geographic factors inhibit the movement of project staff. - They also influence disease risk perceptions and willingness to adopt prevention technologies.	- There is often a seasonal window of opportunity for interventions to be most effective. - Intervention terrains cannot be conceptually uniformly. Rather, special attention needs be given to priority social groups and high-risk epidemiological areas. - Seasonal livelihood changes and human migration patterns need to be accounted for.
Social Difference and Community Agency	- Adoption of health technologies aligns with higher material and social capital. - Socio-economic variations within districts have important consequences for effectiveness. - Motivation to participate is driven by fear/concern for the disease, level of input requested, community cohesion, conceptualization of benefits/value and how the intervention relates to the priorities of everyday life. - Local people interpret and evaluate risk communication according to experience, tradition, prior biomedical knowledge and source trustworthiness. - Village leaders play a major role in translating intervention goals to local idioms and interests, through forms of influence and authority. - There are tensions between people who participate and those that resist the intervention, but resistance can be a form of legitimate grievance to wider processes of marginalization and lack of health services. It can also be the cause of a deliberate and rational weighing of the risks, benefits and requirements for prevention, something that is influenced by disease epidemiology and transmission pathways. - Higher levels of gender equality tend to translate into more successful interventions. - Local leaders sometime use punitive measures to enforce compliance, which can be socially harmful. But social pressure could also be positive.	- Identify geographical locations and social groups that are more likely to be enthusiastic about the intervention and begin implementation with these groups. - Some areas will have stronger local leadership. These leaders could be engaged in implementation efforts in other areas by having them lead learning workshops and help with M&E systems. - Incorporate local experiences, idioms and explanatory frameworks into risk communication. - Use trustworthy sources to disseminate information and engage local communities. - Monitor the emergence of punitive and coercive efforts that may be used by local leaders to enforce interventions and stigmatize the poor.
Strategies and incentives of field staff	- Field staff often do not implement strategies in exactly the same way that planners tell them. This can be good (local innovation) or bad (not following guidelines). - Volunteers and other key staff are often selected based on political patronage, and not necessarily their ability. Some field staff may not be competent enough for the job. They may lack essential knowledge to pass on to community members or not have enough skills to organize the fieldwork and record data. - Interventions can disrupt routine care and outreach, and sometimes take resources away from other activities. - Field staff operate within a fragile system of resource constraints, often with low morale. - Field staff have expectations and professional goals, and these influence their motivation and dedication.	- Strong staff management and regular evaluations and field checks are important. - Clear expectations, resources and guidance need to be provided to field staff. - Planners cannot expect that field staff will use existing resources for things like travel and communication. Field staff need to be adequately resourced and motivated. - Small incentives are important elements of a staff management system, as are regular re-fresher trainings and team meetings
The socio-materiality of technology	The characteristics of intervention technologies mediate adoption, delivery and use patterns: - For insecticides, this included things like smell, color, packaging, residual period and mode of action, as well as spray equipment. - For latrines, this included access to building material, designs, tools and their interaction with weather, insects and soils. - For vaccines, this included side effects, cold chain equipment, syringes, ability to restrain dogs.	- Understanding how people perceive and use the intervention technology is very important, and should be used for plan and inform delivery strategies and approaches. - Sometimes small changes to the packaging or other characteristics of the technology can have a positive influence.
The governance of interventions	- Past policies influence current implementation. - National governance reforms (especially decentralization and liberalization), past intervention histories and global policy contexts influence the behaviour of district management staff. - Intervention narratives often predetermine the course of events and can severely limit the ability for staff to adapt. - The expertise and professional interests of staff and planners influences how well the intervention adapts to local contexts and challenges. - Low-cost and low-tech solutions can be very complex to implement. - District-level departments often lack essential management skills and capacities needed for successful implementation. - There are often hidden histories, different norms and even conflict between different stakeholders and organizations. - The lack of involvement of field staff and district-level management in the planning process leads to a lack of ownership. Together with time-bound targets this creates a culture of poor accountability and a lack of real dedication to the success of the program.	- Incorporating a broad group of stakeholders in the planning process can help anticipate challenges and create a stronger sense of ownership. This should include civil society and social scientists. - It is important that barriers to collaboration between different stakeholders are identified early on and effectively addressed. - Budget allocations in large-scale programs should account for district-level variations. - More attention should be given to data collection, analysis and the use of data for program planning. Learning from communities and field staff is key to effective interventions. - Systems of M&E should balance budget priorities with the need for actionable data. - Plans should include strong follow-up management training, support and oversight to district teams. - Donors and financing partners should be open to adaptations and consider the broader health system context, including incorporating new targets that may seem outside the initial scope of work.

To be clear, budget constraints are an important part of ineffective interventions. For example, there would have likely been a different outcome had SOS continuously subsidized insecticide distribution and funded the private veterinarians for continued social mobilization in endemic villages; the budget for rabies vaccination ensured greater capacity to vaccinate in remote areas; and CLTS paid their volunteers and enrolled the support of other stakeholders through direct financial incentives. Money alone could not have solved all of the barriers to change discussed above, but it would have certainly helped. In an ideal world, program planners would get ample operational budgets. The reality is otherwise. Budgets are also stretched from the start, because those who administer and write the grants know that making big promises (for example, to cover large terrains), sometimes beyond what is really tangible, is what can secure the money from international and domestic political and scientific constituents. They also, of course, want to extend their ambitions for positive population health over as large an area as possible.

These realities aside, there are also lots of ways to better use existing resources. The art of project management is to ensure success in the face of limits–a balancing act.

As I have argued, one major shortcoming of current intervention approaches, whether more top-down (the WHO rabies vaccination program), participatory (CLTS in Zambia) or public-private partnerships (SOS in Uganda)–is the lack of a critical praxis (dialectical movements between reflection and action) embedded within project planning. M&E systems are often weak. Funds are stretched, and/or used in sub-optimal ways. Existing ways of doing things go unchallenged. Hierarchies govern over best practice. Inertia and fatigue sets in. Time is short. Planners spend more time looking upwards to donors than downwards to recipients. The challenge of establishing delivery systems means that community engagement is sidelined. Learning about the details gets pushed to the side.

Proactively learning about the process of implementation, using a framework that is attuned to the forensic details of the process and context itself, may be one *hopeful* antidote to the placidity of this daily-grind. Seeking out information on the terrain of implementation, the agency of local communities, the strategies and incentives of field staff, the socio-materiality of technology and the challenges of governance should, so I hope, assist in promoting interventions as a process of, what Biehl and Petryna [54] have ingeniously called, the task of “endlessly tinkering” intervention techniques and strategies on the ground. An intervention is never a completed process. It is always evolving and changing. It demands revision. It demands an ethical and moral engagement.

Such an orientation should take place at multiple levels, including with local managers and field staff, and with the methodologies and tools of implementation itself. This should include socio-anthropologists communicating more effectively about what they can bring to the table, and be assisted in doing so by promoting a culture of problem solving, of constructive criticism, critique and adaptation [55].

In a recent book, *Reimagining Global Health*, Paul Farmer and colleagues [56] argued that global health needs to take a *biosocial approach* committed to equity and social justice. Many would agree; but the problem is, as the authors note from experience, that “no one sets out to ignore equity…the way we frame issues of causality and response typically fails to give it due consideration” [56]. This gets to the heart of how important our perspectives are in shaping our actions. One small way forward, I think, is to think more critically about what interventions are, what implementation entails and the nature of social action across diverse contexts. To this end, social science, in its ideal form, can offer us what Flyvbjerg [57] has called a “practical wisdom.” Such an approach has much to offer the science of global health delivery, including the control of neglected tropical diseases.
